# Knowing How

**Published:** 1909-09-15

**Authors:** D. M. Matteson


### KNOWING HOW.

BY D. M. MATTESON, D.D.S.

Read before the Michigan Second District Dental Society, April, 1909.

In order to gain success we must have failures, and all feats of skill so far as they are practicable are possible and easy if the operator knows how. I will illustrate this by the synopsis of a story which appeared in the "Saturday Evening Post" a few weeks ago, entitled "The King of Diamonds."

One morning a prominent diamond dealer in New York, received a small package unregistered, and containing no signature to show from whence it came. On opening it he found it to contain a diamond of about six carats in size. He called his diamond expert who examined it and proclaimed it perfect. He was amazed and mystified that a stone of such value and beauty should be sent to him in such a careless way. Later he consulted another prominent dealer—a German— who was considered an expert. He was still further mystified to find that he also had received an exact replica, in the same mysterious way. It developed that three other leading merchants of the city had received similar gems, each one an exact replica of the other, five perfect diamonds larger than any diamonds in America and more perfect than any diamond in the world. A few days later the first merchant received a note telling him that the sender of the diamond would meet him and the other four merchants at the first merchant's store at 3 P. M. on a certain day. Promptly at the hour the mysterious one appeared. Apparently he was a young business man of New York. After meeting all present he said that he had an unlimited supply of diamonds. This statement he proved by producing a number of replicas the exact size and cutting of the largest diamonds in the world and also about $3,000,000 of diamonds of all sizes and colors known to the diamond world. He then made them his proposition, which was, that he would furnish them $100,000,000 worth of diamonds at half the market price per carat, these were to be produced and paid for within one year. He then gave them one week to consider his proposition, advising them that if they did not accept he would flood the market. A series of circumstances developed the fact that the diamonds were manufactured from pure carbon extracted from brown sugar. The originator of the process was killed by a tramp and the exposure followed—The young man who represented the manufacturer took the five merchants to the abandoned coal shaft where the old man carried on the manufacture and explained the process which was by means of a gigantic cube of bifurcated steel in the center of which was an electric furnace, running clear through the cube was a two inch hole, the exact size of the bore of a cannon, stationed on each side and so mounted that as you looked through the open breach of one cannon the bore seemed continuous clear through the cube of steel and the 2nd cannon beyond. By means of the electric furnace the carbon was heated to half the sun's temperature and then the cannon fired by an electric spark caused a compact of about six thousand tons pressure; the cube was immediately opened and the furnace while hot was dropped into water. The innermost contents was taken out and this was a diamond. Said the first merchant, "you have shown us exactly how to do this, why can't we do it?" The answer was, "you can do all this, but the mind that held the secret of extracting pure carbon from brown sugar was the mind of the old gentleman who was killed and the secret died with him." Said the German expert, "The Alchemist tried for three thousand years to manufacture a piece of gold as big as a pin head and failed, and this old man with a little concrete and brown sugar made a diamond as big as a biscuit."

A few years ago I attempted to make two bridges for a gentleman, an upper and lower, both on the right side and extending from the cuspids back to the 2nd molars. I prepared the teeth as I thought they should be. The upper cuspid for a Richmond crown, all the others gold crowns, after taking the impression with crowns in place and getting the models, I assembled the teeth and invested them, heated up the case as best I could with the burner I had, cut solder into small pieces and placed on the piece and proceeded to solder. I used borax for a flux—*lots of it*—I threw the large blow pipe flame on the investment for a long time then turned it on the solder and after a time it melted. After cooling and cleaning the investment off, I found that I had burned the gold crowns so that they were useless and had checked nearly all the facings; I called in my patient and went through the ordeal of making new crowns—took my impression with them in place; assembled the two pieces again—invested, heated them up, and again I spoiled them —I want to tell you that my chances for eternal salvation were all gone that night I came to the conclusion that I did not know how to solder a bridge—Reluctantly I called in my patient for the third time prepared new crowns, took the impression and sent them to a laboratory. They returned O. K., looked nice, no crown burned, no facings checked and they fitted. What was the reason I couldn't do this. I took these old bridges and experimented with them and found that I could not get the solder to flow toward the crowns. I tried this on the Richmond crown with the same result. My conclusion was that I did not get heat enough on the investment before turning it on the solder. Every school boy or freshman ought to know that. So I got a burner that would throw a powerful gasoline flame from below on the investment, tried it and was surprised how easily the investment was heated to the proper heat—the backings and cusps a bright cherry red. I turned on the blow pipe flame and the solder flowed quickly. Before that time I never knew when I was going to make a successful Richmond crown and I came to the conclusion that the only reason I ever had made them successfully was due to bull-headed luck, for I certainly did not know how. Little points picked up later at conventions, principally, made the flowing of solder still easier. Now I use Peso's liquid flux—and cut the solder into one strip, dip it into the liquid and heat it enough so the flux will stick. When the investment is hot the gold crowns—backings show up a bright cherry. I take this strip in the pliars, iridio platinum on the ends, hold it where I want the solder and turn the flame on it as it flows, keep placing it and when that is gone pick up another and so on, when through, place a crucible or ladle over investment and turn down the flame; I generally leave it for half an hour. Another bridge I made of replacable teeth, the anchors being Richmond crowns on the left cuspid and central and right lateral, the bridge extended from the left 2nd molar to the right 2nd bicuspid, in removing from the articulator I broke the model, I put it together as I thought right and soldered. but when I put it in place one central stuck down about an eighth of an inch too far. Immediately came back to me a feat I had seen done by that genius whom at least the older members here knew, Dr. Dorrance. He had a case set up ready to invest, he then painted a little oil over the labial surface of the teeth and then put a thin coat of plaster over the labial surface of the whole bridge. When it was hard he picked it off and saved it for an emergency, and the emergency materialized as I remember, because he broke the investment and had to use this emergency piece. Had I done this would I have saved a whole day's work.

I had just one experience with soldering a gold bridge in an electric furnace. The solder did not flow and when I finally took it out the facings were the most beautiful pea-green you ever saw. I never knew why it happened so, but I never tried it again.

DISCUSSION.

Dr. C. J. Lyons: I can appreciate the difficulties Dr. Matteson has encountered as I made some of the same blunders. I find that I am inclined to use an excess of flux and am not patient enough to wait until the invested bridge is sufficiently heated to permit effective use of the blow pipe. I get best results by preparing my piece so that it will take the solder readily; All joints should be in close approximate contact; the solder should be properly made, and scraped to a clean bright surface, and then cut into suitable pieces and placed on the joint so that it will not be displaced in the heating process: The solder is best kept ready to use by keeping it clean and cut in powdered borax, which has been calcined, the invested piece should be carefully heated to a dull red before the blow pipe is applied for soldering, and the solder should only be fused when the parts are hot enough to receive it.

DR. E. L. WHITMAN: I find that it is very necessary to use an investment that will stand the fire without checking. I have had the best success with a material which I get from the Dental Supply houses, called Tenax, I mix it nearly as thick as I want it and then add all the plaster of Paris I can and invest.

DR. W. D. REED: I have never found better investment material than equal parts of hard coal ashes and plaster of Paris.

DR. F. WINCHESTER: It is easy to do things when you know how. I once tried to vulcanize a tooth to a celluloid plate. I shall never do that again. I have learned from experience that it is not only easy when you know how, but that it is most profitable to know things. I use for investment material dry potters clay from the tile works, mixed with two-thirds plaster. I prefer the liquid flux to the powdered, as it sticks better and the solder does not fly off so badly when the heat is applied.

DR. MATTESON: I will say that I am now using the investment material marketed by Dr. R. C. Brophy and have good success with it.

DR. M. L. WARD: The Brophy investment contains silica and plaster of Paris. One of the strongest investments I know is Dr. Peso's which is made of equal parts of bird gravel and plaster of Paris. It is not very pleasant to handle, but it will not shrink or crack. Any coarse sharp silica and plaster will make a strong investment, but we must expect that all investments will shrink some, especially, when great or long protracted heating occurs. There is an art which must be learned in soldering. The piece should be heated to the soldering point and the solder melted quickly by a proper flame. The blow pipe should have a perfectly smooth, round opening that will give a smooth, round flame, and the air should only be forced hard enough to oxidize the flame to its maximum heating capacity short of a reduction heat. A good test is that there should be no appreciable noise at a distance of six or eight inches from the ear, the flame should be blue with the inner yellow heat cone distinct. When soldering pure gold or platinum put the borax only on the solder. If using alloyed gold I prefer to put dry borax on the backing and in the joints. It is a good plan to cut the solder into long strips and dip it into liquid borax and hold with a pair of soldering tweezers over the joint to be soldered and then melt it with a blow-pipe until the contour restoration desired is made. In bridge work solder the dummies together first and then fill in the contour.

Dr. N. S. Hoff: In my experience I have found that many dentists not only don't know how to do the kind of work under discussion, but they have no proper equipment for this kind of work. I visit many offices in my travels and find most of them fairly well equipped for operative dentistry. But when you step into the laboratory you find a junk room with the most meager equipment; a vulcanizer an old worn out foot lathe, a half doxen old battered impression trays, a double ended vulcanite file and two or three very dull vulcanite scrapers. If there is a blow pipe it is one of the kind that you can hear for a block when it is in action, it is not one of Dr. Ward's silent ones, and everything else in the laboratory is of the same shiftless makeup.

Now how can any one expect to do business successfully with such a backing as that! He can't make an artisan of himself in such an environment no matter how much latent talent he may possess or how persistently he may labor. We must get better laboratories if we are going to do successfully the kind of work that is nowadays popular and demanded. Fine operation outfits should not be set aside or ignored but we are changing our methods of repairing the ravages of dental caries, and it looks as though the laboratory was coming to be the most essential feature in an office equipment. It is not necessary that we should buy everything on the market, but a complete modern laboratory equipment ought to be a part of every dental office.

Dr. J. W. Lyons. I think most of us are inclined to slight the mechanics of our practices. It takes time and deliberation to make a successful mechanic. You can't hurry a vulcanite plate through the making without blundering at some important stage with an unsatisfactory result which a long suffering and ignorant public seems willing to endure. Don't hurry or get nervous about this kind of work, especially, for it almost certainly means a compromise and unsatisfactory result. If you have so much to do that you can't do this work as it should be done, turn it over to some dentist whom you know will give it proper attention. Arrange your work so you can do it orderly and well, charge more for your services and don't work so hard, take vacations often and keep in good health. There is a greater chance for most of us to excel in the mechanical aspect of our profession than in the scientific or professional, then lets not only learn how to do things but prepare ourselves to do them well as possible.

Dr. G. W. Baylis: The advice for better equipped laboratories is timely, but it seems to me that good work can be done with a few well selected tools and better should one know how to most effectively use the few that he already has. I have seen some fine work that was produced in the laboratory of a genius whose laboratory was really not equipped with modern appliances of any sort.

DR. WARD: Continued fusing of the solder will eventually fuse the backings, alloys are quickly formed under the high heats. Melt it to place and remove the flame at once and let it cool.

DR. REED: I solder the dummies first and then unite them. To save money I sometimes use silver as a filling material, coin silver.

